# Foraging connections: Patterns of prey use linked to invasive predator diel movement

**DOI:** 10.1371/journal.pone.0201883

**Published:** 2018-08-15

**Authors:** Cora A. Johnston, Erin E. Wilson Rankin, Daniel S. Gruner

**Affiliations:** 1 Biological Sciences Graduate Program, University of Maryland, College Park, MD, United States of America; 2 Department of Entomology, University of California-Riverside, Riverside, CA, United States of America; 3 Department of Entomology, University of Maryland, College Park, MD, United States of America; Dauphin Island Sea Lab, UNITED STATES

## Abstract

Invasive predators can profoundly impact native communities, especially in insular ecosystems where functionally equivalent predators were evolutionarily absent. Beyond direct consumption, predators can affect communities indirectly by creating or altering food web linkages among existing species. Where invasive predators consume prey from multiple distinct resource channels, novel links may couple the dynamics of disjunct modules and create indirect interactions between them. Our study focuses on invasive populations of *Eleutherodactylus coqui* (Anura: Leptodactylidae) on Hawaii Island. Coqui actively forage in the understory and lower canopy at night but return to the forest floor and belowground retreats by day. Recent dietary studies using gut contents and naturally occurring stable isotopes indicate higher than expected consumption of litter arthropods, which in these Hawaiian forests are primarily non-native species. We used laboratory studies to observe diurnal and nocturnal foraging behavior, and experimental field additions of C_4_ vegetation as a litter tracer to distinguish epigaeic sources from food web pools in the C_3_ canopy. Lab trials revealed that prey consumption during diurnal foraging was half that consumed during nocturnal foraging. Analysis of δ^13^C isotopes showed incorporation of C_4_ carbon into litter arthropods within one month, and Bayesian mixing models estimated that 15–25% of the carbon in coqui tissue was derived from litter sources. These results support recent findings that *E*. *coqui* are not quiescent diurnally but instead actively forage. Such activity by a mobile invasive predator may introduce a novel linkage that integrates detrital and foliar resource pools, potentially distributing influences of invasive litter arthropods through the broader system to amplify impacts on native species.

## Introduction

Biological invasions are major drivers of global change that disrupt species interactions [1–3]. Invasive predators in particular, mediated by their diet in invaded communities, may alter and degrade trophic systems and compromise ecosystem stability. Dietary plasticity and prey switching behavior to exploit multiple prey species that vary spatially and temporally are key traits of successful invasive predators [4–7]. The capacity to exploit other nonindigenous taxa, which often possess traits fostering rapid population growth in their introduced ranges, may support elevated numerical responses and higher population densities of predators [8]. Accordingly, resource subsidies from co-invasions may lead to stronger impacts of invasive predators on native prey species through the mechanism of apparent competition [9]. An integrated understanding of predator diets accounting for all resource pools is therefore required to predict the direct and indirect impacts of invasive predators on native fauna. This need is particularly urgent on isolated oceanic islands, in which invaded communities host a high proportion of endemic species lacking evolutionary histories with functionally similar predators [10–12].

The invasion of the insectivorous frog, *Eleutherodactylus coqui* (Anura: Leptodactylidae; hereafter “coqui”), into the Hawaiian Islands in the 1980s has raised concerns about its impact on Hawaiian forest communities [13–16]. On Hawaii Island, coqui are now an abundant top consumer in low- to mid-elevation forest communities that lack an evolutionary history with amphibian predators. Coqui foraging patterns depend at least in part on diel movement. Territorial in nature, coqui shelter in leaf litter and underground cavities by day and then migrate vertically to forage and mate in the understory, moving an average of 200 ± 95SD cm per night [17–20]. Beard and colleagues [21] estimated that dense invasive populations of coqui may consume 690,000 arthropods per hectare per night in the forest understory on the Big Island of Hawaii. Increasing observational evidence from their invaded range suggests that, unlike in their native range, coqui in Hawaii forage on litter prey, including abundant exotic amphipods, isopods, ants, roaches, and myriapods [20,22,23]. In Hawaiian wet forests, endemic species represent a higher fraction of arthropod communities in the foliage of native trees, whereas epigaeic communities in the litter layers contain more exotic species [24,25]. Thus, coqui’s relative consumption of foliage versus litter arthropod prey is likely to mediate their effects on native prey, relationships to exotic forest arthropods, and the structure of the food web [26].

Dietary gut content and stable isotope analyses have documented litter-based foraging by coqui where they have invaded Hawaii [22,23,26]. This finding was surprising because it may indicate a major shift in the use of resources in their introduced range as compared to populations in their native range, where coqui are considered diurnally quiescent [19]. However, we still lack spatially- and temporally-explicit quantitative estimates of the relative importance of ground- and canopy-based arthropod resources, because attributes of the system limit inferences from observational studies alone. For instance, coqui are rarely collected or observed foraging during the day due to sheltering, often belowground, in secretive refuges. Reports that the coqui diet is comprised of foliage-based communities in its native Puerto Rico were determined from samples collected at 06:00, when–due to 8–12 hr gut retention time–gut contents likely reflect nocturnal prey consumption [19], whereas gut samples from Hawaii containing litter prey were collected closer to 20:00, likely reflecting diurnal consumption [22]. More recently, Wallis and colleagues [23] found that coqui contained more prey items when collected in the morning than evening, suggesting greater nocturnal foraging, though diets were still dominated by litter arthropods. Beyond the challenges of temporal activity patterns for field sampling, studies of gut contents are further limited by taxonomic resolution, especially in instances where digestion resistance biases measurements of prey use towards those with robust body parts [22,27] and where some arthropods occupy multiple forest strata, limiting inference on prey origin.

Stable isotopes are an additional tool useful for identifying prey sources and partitioning dietary composition [28]. For example, Pringle and Fox-Dobbs [29] used isotopic mixing models to infer the relative contributions of canopy- and ground-dwelling arthropods consumed by predators. This was feasible because plants with C_3_ and C_4_ photosynthesis (trees and grasses, respectively, in their system) yield distinct isotopic signatures of carbon (δ^13^C) that propagate to consumers. However, Hawaiian forests are dominated by C_3_ plants, which makes foliage- and ground-based food web sources indistinguishable with naturally occurring carbon isotopes. One powerful experimental approach is to use enriched C_4_ litter to introduce an effective tracer that distinguishes litter- from foliage-based prey sources [30].

Experimental resolution of temporal and spatial prey use can provide a more complete picture of coqui’s impacts on Hawaiian forest ecosystems. Here, we tested coqui use of litter and foliage resources by conducting 1) captive feeding trials in lab mesocosms to assess activity patterns and prey use and 2) stable isotope C_4_ tracer addition plots to experimentally parse the relative contributions of litter and foliage arthropods to coqui diet [29,30]. Our goals were to establish the occurrence and relative importance of diurnal foraging, and to estimate the contribution of litter arthropods to coqui diets. Coqui likely consume prey from all prey pools encountered, as do many generalist opportunistic foragers [4,18,22]; however, spatial and temporal activity habits likely mediate how much of each prey source is consumed [23,31]. Experimentally testing the timing and location of foraging can help determine how coqui interact with foliar and litter-based food webs and determine whether diel movements link discrete and differentially invaded food web communities. We predicted that, through daily vertical migrations, coqui interact with both understory and litter prey pools, potentially altering linkage between these spatially discrete food web compartments. We also used coqui foraging patterns to posit a series of likely direct and indirect routes of impact on recipient Hawaiian forest food webs.

## Methods

This study was conducted on the Island of Hawaii, USA, where invasive *Eleutherodactylus coqui* have reached unprecedented densities of up to approximately 910 individuals per 100 m^2^, with vast potential to alter prey communities [21]. We conducted lab-based feeding trials and isotope field experiments on the Big Island of Hawaii in July and August 2011.

### Feeding trials

To determine diel variation in foraging activity, we conducted captive foraging trials. Coqui were hand-captured in forests of Lower Kaumana at 165m elevation and held for 12 hours with shelter and water but no food to ensure that prey recovered from gut contents were consumed during feeding trials (8–12 hour gut retention time [19]). Each coqui was used in only one trial (day or night). At dawn (day trials, n = 12) or dusk (night trials, n = 12), each coqui was placed in an individual 60 cm x 40 cm x 45 cm translucent plastic tank containing a small water dish, cleaned leaf litter, and vegetation for vertical structure. Eight size- and mobility-matched arthropod prey were then added to each tank: four amphipods (Talitridae: *Talitroides tapitotum*) collected from forest litter and four small ants (Formicidae) collected from foliage were used to represent the two most common prey items in coqui diet studies [13,22,23]. The same prey types and numbers were added in each trial, leaving time of day as the primary variable. During the 12-hour study period, each bin was covered securely with two layers of fine screen, kept in ambient shade conditions, and misted frequently with rainwater to maintain suitable moisture levels. Prey survival rates were 100% when retained in tanks over 12 hours with no coqui present (n = 4 trials), thus we assumed any arthropods missing from coqui trials were eaten. At the end of feeding trials, tanks were inspected for remaining arthropods and coqui were recovered, euthanized, and dissected for gut contents to confirm presence and number of prey consumed. All coqui capture and euthanization in this study was designed to minimize suffering; the protocol was approved by the University of Maryland Institutional Animal Care and Use committee (Protocol Number: protocol #R-11-22).

### Isotope experiment

To estimate the relative contribution of litter versus understory arthropods in the diets of foraging coqui, we established *in situ* experimental plots where natural C_3_ leaf litter was replaced by C_4_ litter gathered locally. Alternative photosynthetic pathways result in distinctive carbon isotope discrimination in C_3_ versus C_4_ plants [32], so the less depleted C_4_ litter (less negative δ^13^C) can be used to trace the litter food web in the background of C_3_ forest.

**Fig 1:**
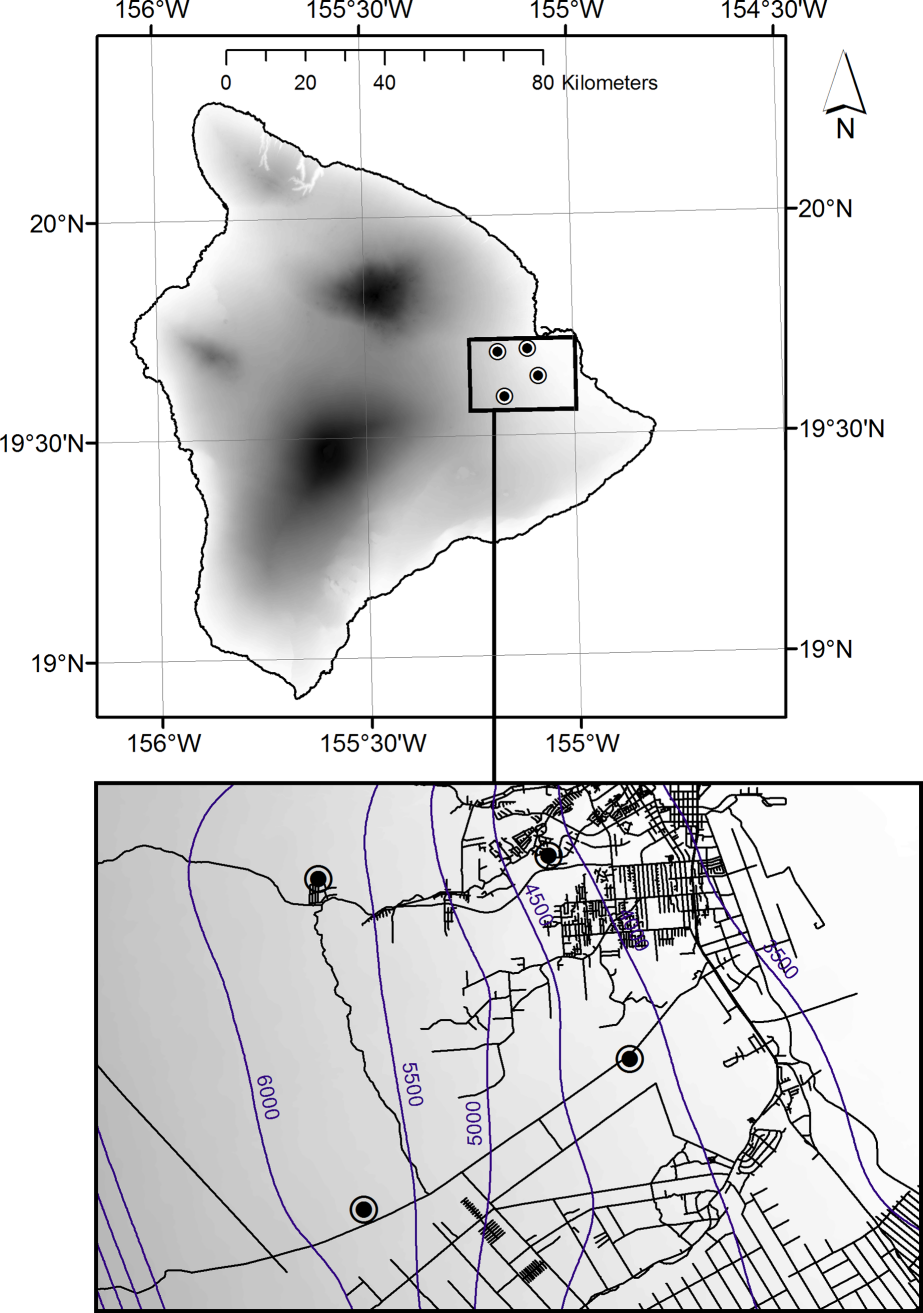
Study sites. Coqui foraging patterns were tested at field study sites (circled black points) on the eastern slope of Hawaii Island, USA. Shading indicates a digital elevation model ranging in greyscale from 0 m above sea level (white) to 4207 m (black). Blue lines delineate mean annual rainfall in 500 mm increments. Reprinted in part from [33] under a CC BY license, with permission from the state of Hawai’i Office of Planning and county of Hawai’i Planning Department, original copyright 2012.

We established experimental plots at four sites in Upper Waiakea and Hilo Watershed Forest Reserves, spanning elevations from 137-740m (Fig 1). Canopy cover ranged from 38–93% (mean ± SE: 73 ± 3%), with 200–600 (348 ± 80) stems per plot. At all sites, coqui densities estimated along line transects [15] (methods as in [22]) ranged from 5–70 (28 ± 9) adult coqui per 100 m^2^ (given adults >25mm SVL, [19]) and were within the range of natural population densities of 6–138 coqui per 100m^2^ reported in Hawaii [21] and 1–24 coqui per 100m^2^ in Puerto Rico [17].

In June 2011, we collected leaves of naturally occurring sugarcane (*Saccharum officinarum* L.) from remnant agricultural patches within 25km of the study site and then dried and clipped them into <20cm lengths. Sugarcane has been cultivated for more than a century worldwide, and its general palatability to arthropod consumers is well documented [34]. In July 2011, we established 21 forested 2m x 2m experimental plots across the four field sites (*n* = 2 to 8 plots per site based on land availability). Plots were not enclosed, but the 2m^2^ plot area encapsulates the daily movements of a coqui, especially territorial males, which move an average of 3–4.5 total linear meters per night including the vertical climb between their litter retreat and calling perch [18]. Coqui do not undergo breeding migrations, and congener individuals remain within 2m of documented locations for over 300 days [35]. We removed leaf litter from each plot and replaced it with 600g of experimental C_4_ litter, based on 150g litter m^-2^ in neighboring forest at 230m elevation [36]. For comparison, the removed forest litter was cleaned of major debris, dried, and weighed (mean ± SD = 509 ± 188 g/plot), revealing that slightly more litter was added than removed (*t* = 2.2, d.f. = 20, *P* = 0.04), though additions were within the range of litter mass removed (200-875g/plot). To account for natural decay and litter fall rates, we supplemented each plot with 120g of additional cane litter after two weeks (15g litterfall m^-2^ per week as in [36].

After one month, beginning in mid-August, each experimental plot was harvested at night between 20:00 and 02:00. To determine prey use based on the C_4_ isotope tracer, we collected coqui, arthropods, and vegetation from each plot for carbon isotope analysis. All coqui present within 1m of the plot perimeter were hand-captured, euthanized, and collected. Leg muscles and the liver were dissected out of each coqui. For plots in which a coqui was captured, foliage clips were taken from dominant vegetation and foliage arthropods were collected with aspirators from a beating sheet and from major stems during a 5-minute, timed search conducted by a single researcher. Foliage clippings were dried and homogenized for isotope analysis. Litter and resident arthropods were sampled by collecting litter within two randomly placed 0.25m^2^ subplots. We extracted litter arthropods from litter samples using Berlese-Tullgren funnels extracted into water; after extraction, a subsample of the resulting dried litter was kept for isotope analysis. Arthropods were pooled into community samples by microhabitat (foliage or litter) for each plot (for a composition overview see S1 Table). All extracted samples were then cleaned and dried to constant mass at 55˚C. Arthropods were analyzed in bulk by microhabitat to achieve sufficient sample mass of small-bodied invertebrates. We ground plant tissues in liquid nitrogen to achieve the pulverization necessary for isotopic analysis. Small hive beetle (*Aethina tumida*) and ohia (*Metrosideros polymorpha*) foliage were included as reference arthropod and plant standards, respectively. Samples were analyzed at University of Maryland’s Appalachian Laboratory using a Carlo Erba NC2500 elemental analyzer interfaced with a Thermo Delta V+ isotope ratio mass spectrometer. Quality control in-house standards were interspersed among the focal samples, and final isotopic values are expressed relative to international standards V-PDB (Vienna PeeDee Belemnite) and Air for carbon and nitrogen, respectively.

### Analysis

We evaluated temporal feeding in captive trials based on the occurrence of feeding (i.e., presence of arthropods in coqui gut) and number of prey items consumed. Mean numbers of prey consumed were compared between night and day trials using a generalized linear model in the ‘car’ package (family = Poisson, link = log) [37] in program R version 3.3.2 [38].

Spatial origin of prey consumed during foraging was estimated from experimental carbon isotopes using Bayesian mixing models. First, isotope signatures for basal (vegetation) and prey (arthropod) source groups were compared with t-tests (after confirming normality) to verify that sources were sufficiently distinct for subsequent analysis. The relative use of prey sources (consumption from litter versus foliage prey pools) by coqui was then assessed with Bayesian mixing models in the ‘SIAR’ package in R using 200,000 iterations and 5,000 burn in [39]. Trophic fractionation factors were not estimated with controlled feeding trials in this study and were not available in the literature for our specific taxonomic groups. Therefore we modeled proportions of source use under a range of reported amphibian and arthropod fractionation values [29,40–49]. We then assessed the sensitivity of model predictions to assigned fractionation. For both arthropods and coqui, we used δ0.5, δ1, δ1.5, and δ2.5^13^C to model the range of carbon fractionation. Further, trophic position influences the number of fractionation steps that occur, which affects the apparent δ^13^C; the effect may be small, but discrepancies can still affect the result of mixing models [44]. Thus, for pooled arthropod samples, we multiplied fractionation values by the approximate trophic position of the component taxa based on literature [40] and taxonomic guilds in litter and foliage samples from our initial site surveys (S1 Table). Assuming trophic steps of 1 for primary consumers, 1.5 for omnivores and detritivores, and 2 for predators, we multiplied litter arthropod fractionation values by 2 trophic steps and foliage arthropods fractionation values by 1.5 [40,45]. To account for uncertainty in fractionation values and dietary assimilation of carbon pools, coqui diets were modeled two ways: (1) based on trophic steps and fractionation from arthropod prey, and (2) based on trophic steps and fractionation from basal plant resources for a more conservative estimate of litter use. Per SIAR specifications, discrimination factors were added to sources rather than subtracted from consumers, allowing us to assign distinct discrimination factors to each source (e.g. based on estimated trophic position among litter versus foliage arthropods).

## Results

### Feeding trials

**Fig 2:**
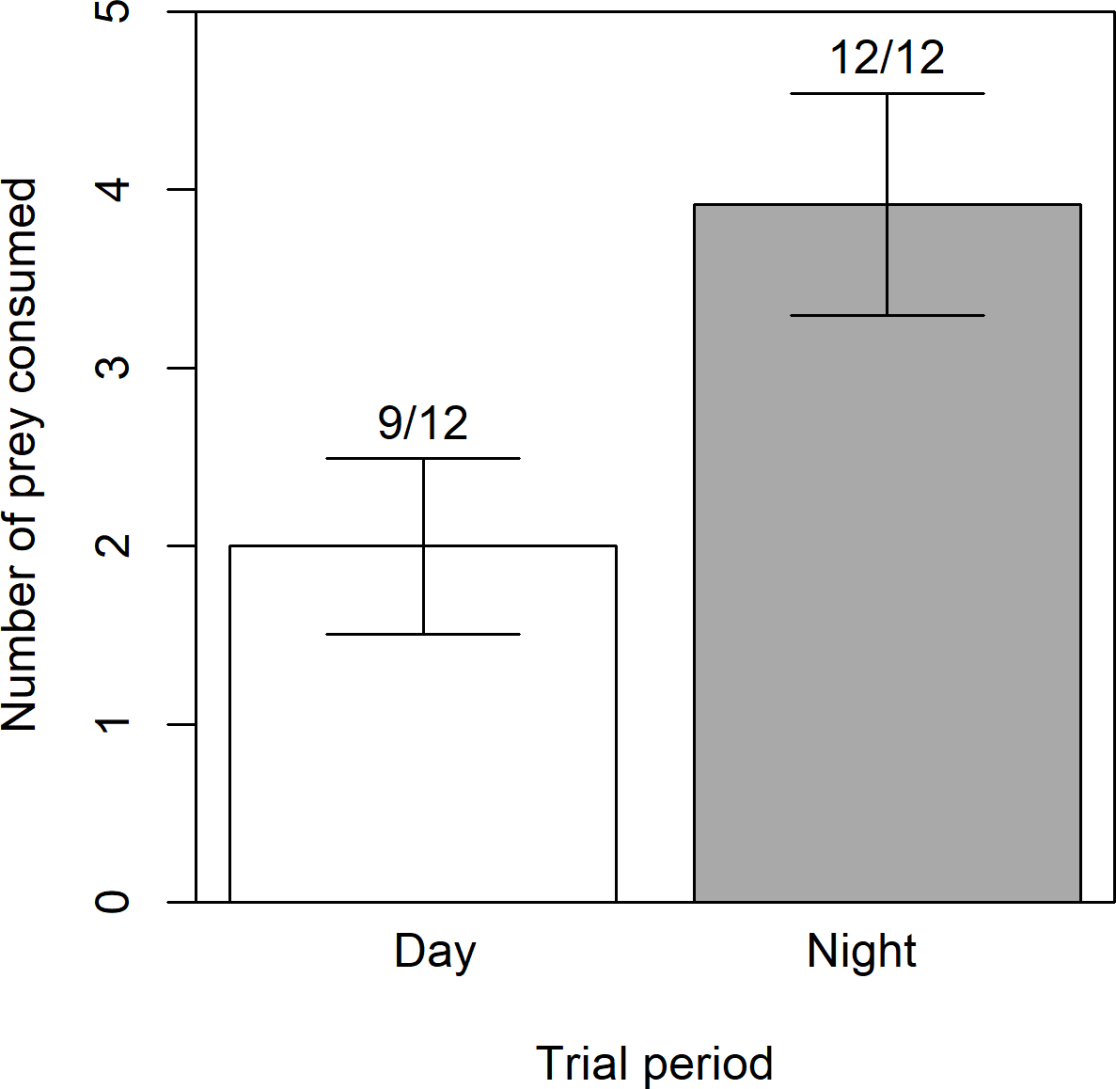
Laboratory tests of temporal foraging. In laboratory foraging trials, coqui consumed prey during both day and night treatments. The average number of prey eaten over 12 hours differed significantly by time of day (n = 12 of each); bars represent one standard error. Numbers above each bar indicate the proportion of trials in which at least one prey item was eaten.

Coqui consumed arthropod prey during both diurnal and nocturnal feeding trials. Coqui consumed at least one prey item in 75% of day trials (*n* = 9/12) and 100% of night trials (*n* = 12/12). Coqui consumed both prey types during both trial periods. Coqui consumed an average of 2.0 arthropods per day trial, while consuming twice that (3.9 arthropods per trial) at night (GLM Likelihood Ratio Test χ^2^ = 7.6, d.f. = 23, *P* = 0.006, *n* = 12 per treatment; Fig 2). Relative foraging rates match temporal shifts in coqui behavior; observations for coqui presence and health during feeding trials revealed that captive coqui were always found under litter or debris during the day, but that activity–including calling, foraging, and movement around the tank–increased markedly at dusk (CAJ, personal observation).

### Isotope experiment

Coqui populations subsided throughout the experimental period, likely due in part to a mid-summer drought (R. McGuire, personal communication); consequently, individual replicates were included only if a coqui was captured within 1m of the plot perimeter. Eight plots (*n* = 1 to 4 per site) contained coqui and were successfully harvested for isotopic analysis. A total of 14 individual adult coqui (11 males, 2 females, 1 undetermined; *n* = 1 to 7 per site) were captured and processed from experimental plots across all sites. Coqui liver and muscle tissues were statistically indistinguishable (t-test, d.f. = 23.6, *P* = 0.5; -25.3 and -25.1 δ^13^C, respectively), but high C:N ratios in liver raised concerns over erroneously depleted δ^13^C signatures in the lipid-enriched tissue, so all subsequent analyses were conducted solely on muscle tissues [50].

**Fig 3:**
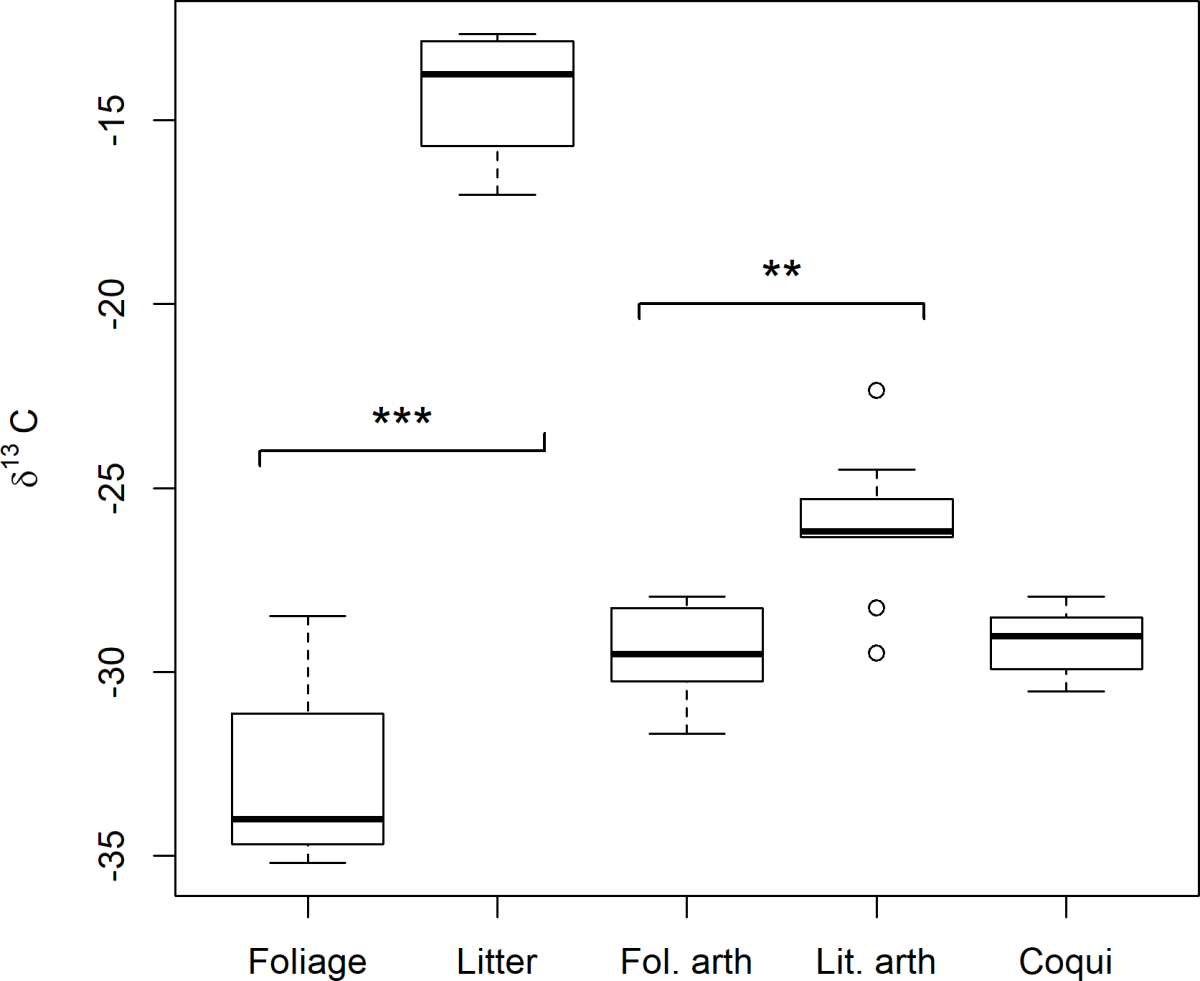
Field study of spatial prey sources. Carbon stable isotope signatures for predatory *Eleutherodactylus coqui*, candidate arthropod prey sources pooled by habitat type (Fol. arth = foliage arthropods, Lit. arth = litter arthropods), and basal producers. The litter was dried C_4_ sugarcane that was added as an experimental tracer to distinguish foraging between litter and foliage prey sources. Brackets delineate source pairs; asterisks indicate statistically distinguishable (*** for *P* < 0.0001, ** for *P* < 0.001) signatures between members of a pair–criteria for use in dietary mixing models.

Based on successfully harvested plots, C_4_ tracer litter was significantly enriched in C^13^ compared to natural forest foliage (mean ± SD: -14.3 ± 1.69 and -32.8 ± 2.55 δ^13^C, respectively; *t* = -16.301, d.f. = 10.2, *P* < 0.0001). Consequently, litter and foliage arthropod prey developed significantly different carbon isotope signatures (-23.0 ± 2.06 and -27.2 ± 1.29 δ^13^C, respectively; *t* = -5.0906, d.f. = 13.6, *P* = 0.00018; Fig 3 and S1 Fig).

**Fig 4:**
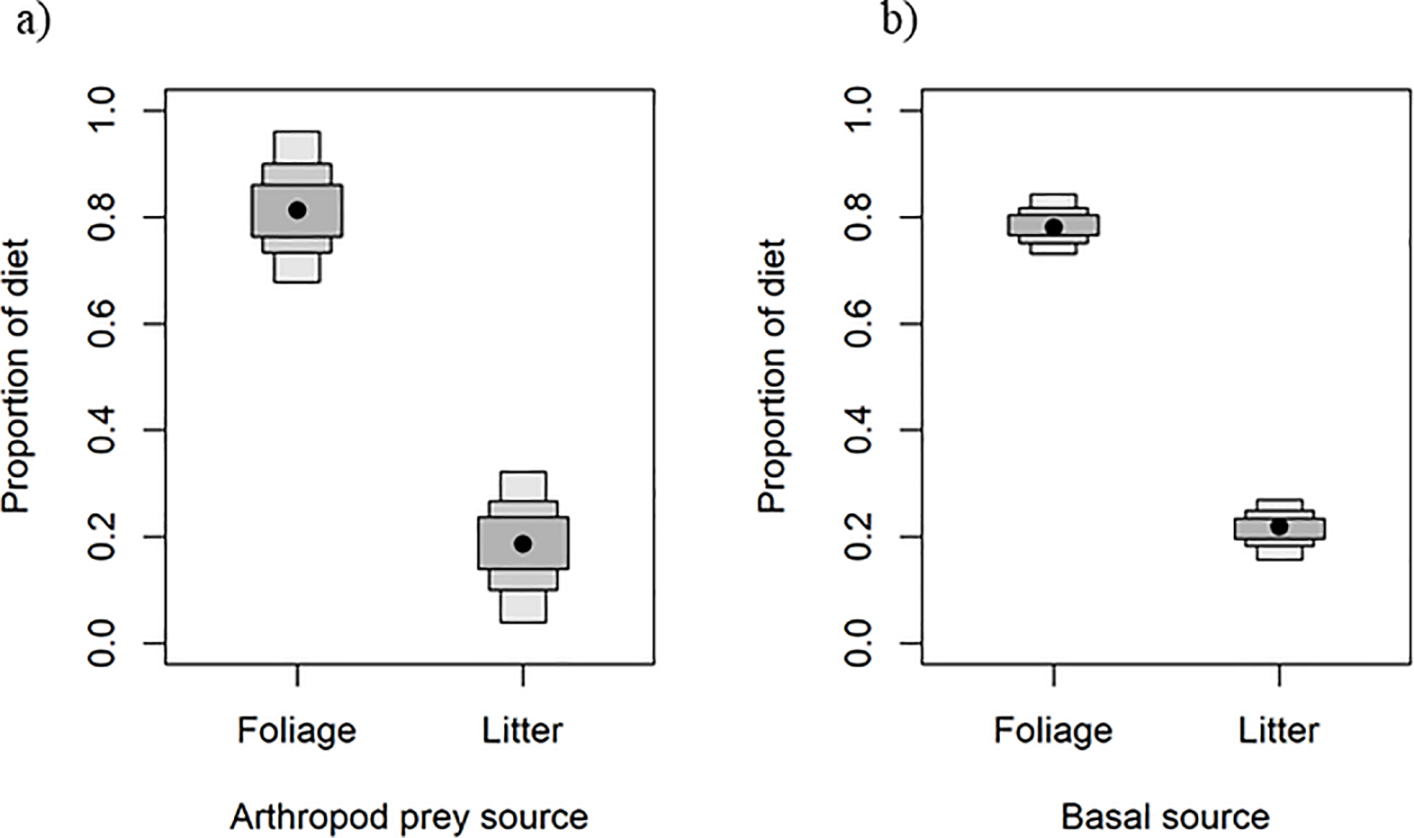
Modeled diet contributions. Bayesian mixing model estimations for the contributions of foliage and litter prey to *Eleutherodactylus coqui* diet based on isotopic signatures of a) arthropod prey grouped by source habitat and b) basal vegetation resources. Basal vegetation sources are naturally occurring forest foliage and experimentally added C_4_ dried sugarcane leaves as a litter isotopic tracer. The trophic enrichment factor of 1.5 –the average of range of values tested–was used in both models. For basal vegetation resource contribution estimates, the trophic enrichment factor of 1.5 was multiplied by one (trophic step to *E*. *coqui*) plus the average trophic position of each arthropod prey group determined from literature (1.5 for foliage and 2 for litter). Black points indicate means and boxes indicate 95% credible intervals for diet contributions: a) 0.82 (0.68–0.97) foliage arthropods, 0.18 (0.04–0.32) litter arthropods; b) 0.79 (0.73–0.84) foliage vegetation source, 0.21 (0.16–0.27) litter vegetation source.

Coqui muscle isotopic signatures were tightly clustered (-25.1 ± 0.86 δ^13^C) and intermediate to litter (-14.3 δ^13^C) and foliage (-32.8 δ^13^C) sources (Fig 3). Across trophic enrichment factors of 0.5 to 2.0, litter contribution to coqui diets ranged from 10–40% (estimated from arthropod prey) or 15–35% (estimated from vegetation basal resources; S2 Table). Across modeling approaches and trophic enrichment factors, SIAR Bayesian mixing models indicated an average resource contribution of approximately 25% litter and 75% foliage across the two modeling methods (mean ± SE litter contribution based on insect prey sources: 24.25 ± 6.54%; based on basal vegetation sources: 25 ± 4.26%; Fig 4).

## Discussion

Previous observational studies in Hawaii indicate that coqui consume litter-associated arthropods and that their guts contain prey items early in the evening, which would be consistent with diurnal foraging [18,19,22]. Here, we experimentally verified that coqui consume prey from both litter and foliage communities. Lab trials revealed that coqui consumed arthropods during both day and night periods, while the C_4_ isotope tracer experiments indicated that litter arthropods significantly contribute to the coqui diet. A clear signal of litter prey is evident in coqui diets despite the spatial and temporal constraints of our study that limit the precision of source contributions. Thus, although coqui forage more consistently and consume more arthropods at night from the foliage, they clearly derive sustenance from both prey sources. Temporal feeding trials and isotope tracer experiments estimate that diurnal foraging and litter sources constitute approximately 25–33% of coqui diet at our sites in Hawaii (Figs 2 and 4), such that this invasive predator is strongly linked to foliage (nocturnal) and relatively weakly linked to litter (diurnal) food web compartments.

### Spatial and temporal foraging patterns

Feeding trials documented the presence and relative rate of diel foraging patterns. Consumption rates during lab feeding trials were within the range of prey per stomach reported for wild-caught coqui: 3.3 in Puerto Rico [18] and between 4.5 ± 3.2 SD (CAJ, unpublished data) and 7.6 ± 7.6SD [22] in Hawaii. Both predator satiation [51] and prey availability [52] can affect foraging rates. Although coqui never consumed all prey offered, predator satiation should not have impacted inference, as presence of foraging activity and relative rate of prey consumption were the main metrics of our study. Moreover, failure to consume all offered prey items indicated that prey availability did not limit foraging rates. Documented rates of diurnal and nocturnal foraging could have been influenced by temporal availability of prey (i.e., prey diel activity patterns; [53,54]; however, prey should have been available throughout trials, because 1) ants are active during the day and 2) coqui preferentially consume amphipods at night in the wild [23]. Thus, relative prey consumption during captive feeding trials indicated that coqui primarily foraged at night, but also consistently consumed prey during daylight hours, despite decreased frog activity.

The C_4_ stable isotope tracer revealed a clear signature of litter prey consumption by coqui (Figs 3 and 4), but the precision of estimated litter contribution is limited by the spatial and temporal confines of our experiment. The two mixing model approaches (i.e. arthropod sources vs basal vegetation sources) were used to bracket uncertainty and ensure that our estimates of diet contribution were robust to two conflicting sources of uncertainty. Due in part to the brevity of the experiment (30 days), assimilation of C_4_ tracer signature may be incomplete for both arthropods (tissue turnover rates of approximately 21 days, [55]) and frogs (turnover of approximately 60+ days, [48,50]). The contribution of litter arthropods to coqui diet may be overestimated if, due to the incomplete assimilation of C_4_ signature by litter arthropods, isotopic mixing models are attributing some of the carbon signature of foliage to incompletely shifted litter sources. On the other hand, litter source contribution may be underestimated due to residual background signal from litter arthropod consumption of natural C_3_ litter before the experiment or to energy exchange beyond plots. Although plots were designed to incorporate the spatial range of daily coqui movement, they were not enclosed and were surrounded by “background” C_3_ litter, which is isotopically indistinguishable from foliage sources. Litter prey movement across plot boundaries or coqui movement and feeding on unmanipulated litter sources outside the experimental plots (i.e., not all litter prey sources have the C_4_ tracer signature) could both contribute to underestimated litter contributions.

To evaluate prey contributions accounting for these competing sources of inaccuracy, we present alternative diet estimates: the mixing model conducted with arthropod prey provides a more liberal estimate of litter use (Fig 4A, S2 Table), while the double-fractionated mixing model using basal litter and foliage vegetation sources provides a more conservative estimate of litter use (Fig 4B, S2 Table). Despite these countervailing conditions limiting certainty and precision of estimated litter contributions, our data converge on similar estimates of litter foraging by coqui during the day. Both models converged on matching estimates of 25% litter and 75% foliage contributions, suggesting that our estimates are robust to spatial and temporal sources of error.

Our estimates of litter contribution to coqui diet based on isotopes are considerably lower than prior estimates from gut contents (60% litter prey [22]). There are several possible sources of this discrepancy. First, gut contents characterize prey ingestion, while isotopes characterize prey assimilation, such that differences between them may indicate discrepancies between volumetrically versus energetically important prey [27]. High digestibility can reduce prey item detection in gut contents and low digestibility can lower the energetic contribution of common prey items. Although we cannot definitively assign litter or foliage origin to arthropod groups, their representation in stomachs of frogs collected for isotopic analysis qualitatively reflect Beard’s prior results (S3 Table) [22]. Second, replacement of forest litter by experimental sugarcane litter could have inadvertently changed the litter invertebrate community, altering prey availability for foraging coqui. Litter quality and nutrient availability can influence epigaeic communities and often differ between native and exotic plant species [56]. In Hawaii, litter invertebrate communities differed markedly between the litter of native *Metrosideros polymorpha* and exotic *Falcataria moluccana* [36]. However, that effect likely originates at least in part from differences in C:N ratio and nutrient cycling that arise from N-fixation by *F*. *moluccana* (resulting in a 4-fold lower C:N ratio [57]). N-fixing and woody species have the greatest impacts on nutrient cycling, which can affect litter communities [56,57]. In this study, however, experimental herbaceous litter and naturally occurring forest foliage did not differ in C:N (mean ± SD: 50 ± 6.8 and 46 ± 5.9, respectively; *t* = -1.06, d.f. = 11, *P* = 0.31). Moreover, Hughes and Uowolo [57] found that changes in litter nutrient cycling and associated epigaeic communities are more likely at the stand level, suggesting that our small-scale manipulation is unlikely to have changed the litter community. Finally, sources of isotopic error likely make our estimates of litter contribution conservative (see prior paragraph). Ultimately, we have experimentally confirmed diurnal foraging and litter prey use, and the quantitative contributions of litter and foliage sources should be tested with longer term isotope experiments, including invertebrate composition surveys in experimental litter and experimentally derived assimilation rates for coqui and arthropod prey.

### Implications: Coqui impacts on food web dynamics

Our experiments confirmed that coqui use multiple prey pools and suggest that invasive coqui could impact Hawaiian forest communities through complex food web networks. Novel predators add links and may modify food web topology, but their interactions with established non-native prey can accentuate their impacts on recipient systems [58]. By actively moving between habitats, an introduced generalist predator can become a centralized node in multiple recipient food webs, connecting previously compartmentalized sub-webs [59,60]. Where exotic prey are not evenly distributed across the landscape, added connections via novel predation linkages across food web compartments may extend non-native impacts through the greater web [61]. Weak links to litter prey may stabilize and subsidize coqui populations, causing apparent competition between the foliage and litter communities [62,63]. Under such a scenario, Hawaiian arthropods may share the burden of coqui predation, thereby buffering against detectable changes in prey communities despite unprecedented coqui population sizes and consumption rates [9,62]. Alternatively, if coqui consumption pathways are inordinately large relative to the diverse, weaker linkages that typically characterize abundant arthropod food webs (like Hawaiian forests; [64,65]), as could be expected for naïve prey encountering a novel predator [5,66], then system stability may be reduced. We show that coqui interact with two food web compartments–strongly with foliage, weakly with litter. Coqui foraging has the potential to increase connectance, decrease compartmentalization, and modify the distribution of interaction strengths–including an increase in the maximum linkage strength–of Hawaiian forest food webs. Given the variety and interrelation of these avenues of influence, theory predicts several potential routes of coqui impact on forest food web characteristics, even in areas where there is minimal evidence of direct prey suppression [15].

### Conclusion

Invasive species are global drivers of change in their recipient communities, where the effects of exotic predators are mediated by their diet composition and foraging patterns [1,2]. Interactions with both native and non-native prey complicate the impacts of exotic predators. Our tests of coqui foraging behavior and resource use in manipulative lab and field experiments provide the first temporally- and spatially-integrated estimates of the extent and potential implications of prey use by this exotic, mobile predator. By consuming both litter and foliage prey, likely the result of daily vertical migration, coqui are likely catalyzing indirect effects of invasive arthropods on native arthropods. This is of particular concern considering the hyperabundance of invasive litter arthropods [24,25,67] in the Hawaiian forest communities that remain a hotspot of foliage arthropod endemism. Accumulating evidence suggests that interactions between exotic species can exacerbate their indirect effects on native species [68]. For example, exotic prey may indirectly increase impacts on native prey by stabilizing or bolstering invasive predator populations (*Vespula spp*: [4], *Rattus spp*: [69]). Additionally, consumption from both litter and foliage-based prey sources suggests the potential for novel or strengthened coupling of detrital and grazer food webs. By consuming prey from spatially distinct communities, *E*. *coqui* may be having more complex effects on Hawaiian prey communities via changes in food web structure and, thus, stability [11]. The diverse foraging patterns of an invasive predator documented here support growing evidence that we need to more carefully consider the indirect effects of species invasions on recipient communities [69].

## Supporting information

S1 Table Arthropod composition by habitat type. Composition (in foliage or litter) estimated from foliage clips and Burlese-Tullgren litter extractions collected from the four survey sites on the Island of Hawaii, USA in July 2011.
(DOCX)

S2 Table Trophic enrichment factor sensitivity analysis. Models run with a range of trophic enrichment factors (TEF) derived from the literature indicate the robustness of diet estimates to applied TEF values.
(DOCX)

S3 Table Coqui stomach contents. Total and proportional abundance of invertebrate prey items from stomachs of 12 frogs collected from experimental plots for isotopic analysis. Stomachs contained a mean of 4.5 ± 3.2 SD items. Prey taxa that made up 10% or greater of total stomach contents are highlighted in bold. Amphipoda and Isopoda are predominately litter-associated taxa; Hemiptera are foliage associated; Hymenoptera may be associated with either.
(DOCX)

S1 Fig **Bayesian mixing model estimates of diet composition** for a) foliage and b) litter arthropods based on a trophic enrichment factor of 1.5 per trophic step. Basal sources are naturally occurring forest foliage and experimentally added C_4_ dried sugarcane leaves as a litter isotopic tracer. Trophic enrichment factors were multiplied by average number of trophic steps per arthropod group derived from the literature (1.5 for foliage arthropods, 2 for litter arthropods). Black points indicate means and boxes indicate 95% credible intervals for diet contributions for a) foliage arthropods (0.82 [0.74–0.90] foliage, 0.18 [0.10–0.26] litter) and b) litter arthropods (0.61 [0.53–0.69] foliage, 0.39 [0.31–0.47] litter). These mixing models of arthropod diet indicate that on average (across fractionation values) litter insects derived twice as much carbon from experimental litter sources as did foliage arthropods.
(DOCX)
