# Supplementary material for: Foraging connections: Patterns of prey use linked to invasive predator diel movement
Source: PLoS One. 2018 Aug 15;13(8):e0201883. doi: 10.1371/journal.pone.0201883 (PMC6093679; doi:10.1371/journal.pone.0201883)
Supplement: S1 Table — Composition (in foliage or litter) estimated from foliage clips and Burlese-Tullgren litter extractions collected from the four survey sites on the Island of Hawaii, USA in July 2011. (DOCX) [file pone.0201883.s001.docx]

**S1 Table**

| Taxa | Foliage | Litter |
| --- | --- | --- |
| Acarina | 1 | 1 |
| Amphipoda | 0 | 1 |
| Arachnida | 1 | 1 |
| Auchenorrhyncha and Sternorrhyncha | 1 | 1 |
| Chilopoda | 0 | 1 |
| Colembolla | 1 | 1 |
| Coleoptera | 0 | 1 |
| Diptera | 0 | 1 |
| Hemiptera | 1 | 1 |
| Hymenoptera | 1 | 1 |
| Isopoda | 1 | 1 |
| Lepidoptera | 1 | 1 |
| Thysanoptera | 1 | 0 |
| Gastropoda | 1 | 1 |
| Megadrilacea | 0 | 1 |
| *Total richness at the resolution identified* | 10 | 14 |
