# Supplementary material for: Foraging connections: Patterns of prey use linked to invasive predator diel movement
Source: PLoS One. 2018 Aug 15;13(8):e0201883. doi: 10.1371/journal.pone.0201883 (PMC6093679; doi:10.1371/journal.pone.0201883)
Supplement: S2 Table — Models run with a range of trophic enrichment factors (TEF) derived from the literature indicate the robustness of diet estimates to applied TEF values. (DOCX) [file pone.0201883.s002.docx]

**S2 Table**

|  |  |  |  |  | % composition of diet items | | | |
| --- | --- | --- | --- | --- | --- | --- | --- | --- |
| Model Type | Consumer | Resource (modeled) | Trophic steps | TEF | % foliage insect | % litter insect | %fol | %litt |
| Frog eats insects | Frog | Insect | 1 | 0.5 | 60 | 40 |  |  |
| Frog eats insects | Frog | Insect | 1 | 1 | 71 | 29 |  |  |
| Frog eats insects | Frog | Insect | 1 | 1.5 | 82 | 18 |  |  |
| Frog eats insects | Frog | Insect | 1 | 2 | 90 | 10 |  |  |
| Frog eats vegetation (via insects) | Frog | Plant | 1.5 foliage invert or 2 litter invert + 1 to frog | 0.5 |  |  | 65 | 35 |
| Frog eats vegetation (via insects) | Frog | Plant | 1.5 foliage invert or 2 litter invert + 1 to frog | 1 |  |  | 72 | 28 |
| Frog eats vegetation (via insects) | Frog | Plant | 1.5 foliage invert or 2 litter invert + 1 to frog | 1.5 |  |  | 78 | 22 |
| Frog eats vegetation (via insects) | Frog | Plant | 1.5 foliage invert or 2 litter invert + 1 to frog | 2 |  |  | 85 | 15 |
|  |  |  |  | Mean | 75.75 | 24.25 | 75 | 25 |
|  |  |  |  | SD | 13.07 | 13.07 | 8.52 | 8.52 |
|  |  |  |  | SE | 6.54 | 6.54 | 4.26 | 4.26 |
