# Supplementary material for: Foraging connections: Patterns of prey use linked to invasive predator diel movement
Source: PLoS One. 2018 Aug 15;13(8):e0201883. doi: 10.1371/journal.pone.0201883 (PMC6093679; doi:10.1371/journal.pone.0201883)
Supplement: S3 Table — Total and proportional abundance of invertebrate prey items from stomachs of 12 frogs collected from experimental plots for isotopic analysis. Stomachs contained a mean of 4.5 ± 3.2 SD items. Prey taxa that made up 10% or greater of total stomach contents are highlighted in bold. Amphipoda and Isopoda are predominately litter-associated taxa; Hemiptera are foliage associated; Hymenoptera may be associated with either. (DOCX) [file pone.0201883.s003.docx]

**S3 Table**

| Taxa | Total abundance | Proportional abundance |
| --- | --- | --- |
| Acarina | 1 | 0.02 |
| **Amphipoda** | 13 | **0.24** |
| Arachnida | 1 | 0.02 |
| Chilopoda | 2 | 0.04 |
| Coleoptera | 4 | 0.07 |
| Collembola | 1 | 0.02 |
| **Hemiptera** | 6 | **0.11** |
| **Hymenoptera** | 12 | **0.22** |
| **Isopoda** | 7 | **0.13** |
| Lepidoptera | 2 | 0.04 |
| Orthoptera | 1 | 0.02 |
| Psocoptera | 2 | 0.04 |
| Unknown | 3 | 0.05 |
| TOTAL | 55 | 1.00 |
