# Supplementary figures and images for: Foraging connections: Patterns of prey use linked to invasive predator diel movement
Source: PLoS One. 2018 Aug 15;13(8):e0201883. doi: 10.1371/journal.pone.0201883 (PMC6093679; doi:10.1371/journal.pone.0201883)

**S1 Figure**

a) b)


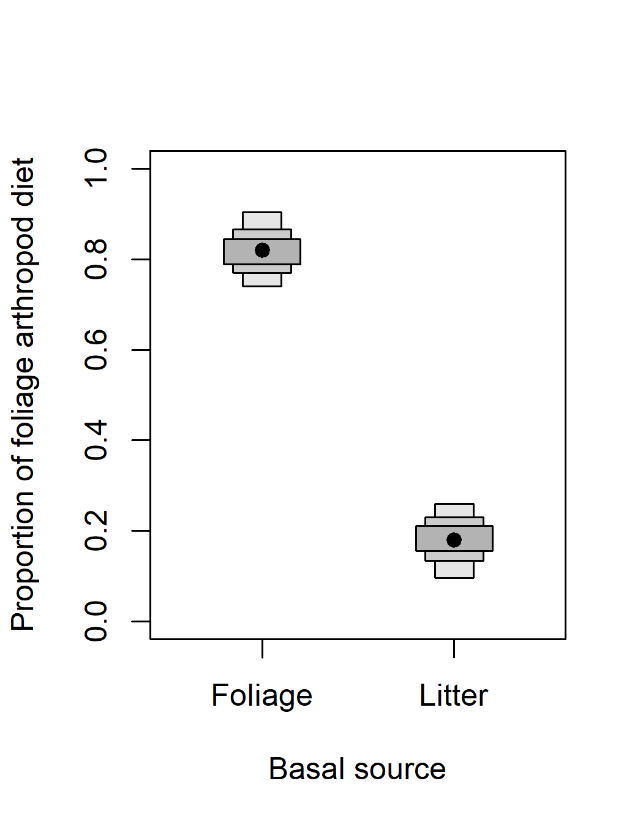

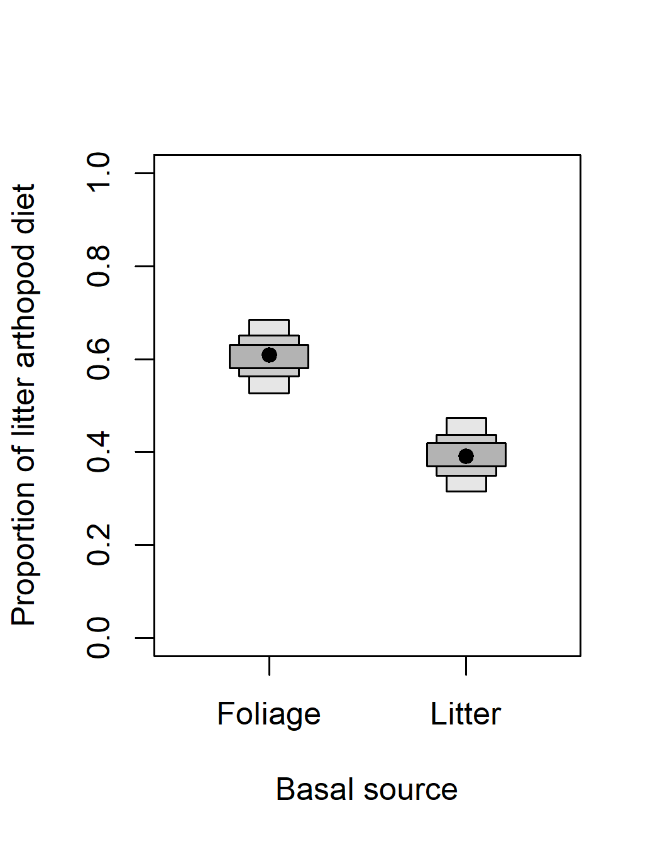

Supplement: S1 Fig — Bayesian mixing model estimates of diet composition for a) foliage and b) litter arthropods based on a trophic enrichment factor of 1.5 per trophic step. Basal sources are naturally occurring forest foliage and experimentally added C4 dried sugarcane leaves as a litter isotopic tracer. Trophic enrichment factors were multiplied by average number of trophic steps per arthropod group derived from the literature (1.5 for foliage arthropods, 2 for litter arthropods). Black points indicate means and boxes indicate 95% credible intervals for diet contributions for a) foliage arthropods (0.82 [0.74–0.90] foliage, 0.18 [0.10–0.26] litter) and b) litter arthropods (0.61 [0.53–0.69] foliage, 0.39 [0.31–0.47] litter). These mixing models of arthropod diet indicate that on average (across fractionation values) litter insects derived twice as much carbon from experimental litter sources as did foliage arthropods. (DOCX) [file pone.0201883.s004.docx]
